# Are we really unconscious in “unconscious” states? Common assumptions revisited

**DOI:** 10.3389/fnhum.2022.987051

**Published:** 2022-10-05

**Authors:** Andre Sevenius Nilsen, Bjørn E. Juel, Benjamin Thürer, Arnfinn Aamodt, Johan F. Storm

**Affiliations:** ^1^Department of Physiology, Institute of Basic Medicine, University of Oslo, Oslo, Norway; ^2^School of Medicine and Public Health, Wisconsin Institute for Sleep and Consciousness, University of Wisconsin-Madison, Madison, WI, United States

**Keywords:** consciousness, unconsciousness, anesthesia, sleep, disorders of consciousness

## Abstract

In the field of consciousness science, there is a tradition to categorize certain states such as slow-wave non-REM sleep and deep general anesthesia as “unconscious”. While this categorization seems reasonable at first glance, careful investigations have revealed that it is not so simple. Given that (1) behavioral signs of (un-)consciousness can be unreliable, (2) subjective reports of (un-)consciousness can be unreliable, and, (3) states presumed to be unconscious are not always devoid of reported experience, there are reasons to reexamine our traditional assumptions about “states of unconsciousness”. While these issues are not novel, and may be partly semantic, they have implications both for scientific progress and clinical practice. We suggest that focusing on approaches that provide a more pragmatic and nuanced characterization of different experimental conditions may promote clarity in the field going forward, and help us build stronger foundations for future studies.

## Introduction

Empirical research is based on the ideal of objective (observer-invariant) measurements and observations of phenomena. Studying consciousness, i.e., subjective experience, or “what it is like to be…” (Nagel, 1974), is a special case, since the phenomena of conscious experience are essentially subjective (observer-variant), i.e., only directly observable from “within” by the individual having the experience. As directly investigating the internal perspective of a system from the outside is believed to be epistemologically impossible—an issue often referred to as the “Leibniz’s gap”—empirical consciousness research depends on inference from objectively observable properties and events, including behavior.^1^ From such research, the common-sense view that our brain is essential for our conscious experience, is supported by a range of empirical studies (Koch et al., 2016; Northoff and Lamme, 2020; Sarasso et al., 2021). Essentially, conventional empirical consciousness science is based on the physicalist presupposition that “there can be no change in the mental states of a person without a change in brain states”^2^ (Pinker, 2003).

Just as in other scientific fields, studying mechanisms of consciousness, i.e., the necessary and sufficient mechanisms distinguishing between unconscious and conscious states, requires a minimal contrast (Revach and Salti, 2021). That is, in addition to examples of truly conscious brain states (states in which it is “like something to be”), we need to identify at least one at least one example of a truly unconscious brain state (a state in which it is “not like anything to be”).^3^ It seems intuitively obvious and uncontroversial that we are conscious during normal, alert wakefulness, whereas brain death with an isoelectric encephalogram (EEG) implies complete unconsciousness. Other states such as deep sleep, dreaming, anesthesia, and different disorders of consciousness (DOC), are considered to exist on some continuum between these two extremes (e.g., Laureys, 2005), and/or are often grouped as either states of consciousness or unconsciousness (implying a dichotomy or threshold, at least in the way it is phrased; see Supplementary material for some examples).

These intuitions have for long shaped experimental research (Searle, 1998) and commonalities in neural dynamics between states classified as “unconscious” (such as slow-wave activity in EEG) have been proposed as potential “markers of unconsciousness” (Gibbs et al., 1935; Posner et al., 2007; Voss and Sleigh, 2007; Casey et al., 2022). However, how confident can we be that these states are, in fact, examples of unconsciousness? From the subjective perspective, only the presence of consciousness is epistemologically accessible (one cannot experience non-experience). And although we can all think of situations where we seem to have been unconscious in the past (what was it like to be you at 4 a.m. last night?), there is a possibility that we might have been conscious but just don’t remember it. Thus, from the third person perspective, while we may have high confidence in inferences about the presence of consciousness in normally awake humans (e.g., based on reports of experience), we cannot have the same confidence in inferences about unconsciousness (e.g., lack of report and behavior; even reports of no subjective experience, may be confounded by memory issues).

What we’ve discussed so far is of course not novel and relates to the “hard problem” (Chalmers, 1995) and the explanatory gap (Levine, 1983), among other philosophical issues (for a more formal treatment see also Kleiner and Hoel, 2021). It has been argued that the above issues can be discounted (for now) and empirical research should instead focus on the “easy” or “real” problems of consciousness to see how far we can get (Varela et al., 2017; Chalmers, 1995; Crick and Koch, 1998; Seth, 2016, 2021). While there is much merit in the “easy” and “real” problems approaches, or working toward establishing the “full neural correlate of consciousness” (Crick and Koch, 1998; Koch et al., 2016), we here argue that common, implicit assumptions about unconscious states ought to be critically re-examined.

## Studying unconsciousness empirically

Physiological, behavioral, and experiential states such as wakefulness, deep slow-wave sleep, REM sleep with vivid dreams, anesthesia, hallucinations, and DOC, intuitively seem to differ in terms of subjective experience, i.e., “what it is like” to be in these states. Accordingly, much of modern consciousness science is based on the distinction between the experiential content and “level” of such states. In particular, general anesthesia induced by certain anesthetics,^4^ deep slow-wave sleep, and certain DOCs (e.g., coma and unresponsive wakefulness syndrome; UWS), have been studied extensively using a wide range of measurement techniques (e.g., Gibbs et al., 1935; Ferenets et al., 2006; Palanca et al., 2009; Nicolaou and Georgiou, 2011; Casali et al., 2013; Lee et al., 2013; Schartner et al., 2015; Juel et al., 2018). In the last 15 years alone, over 200 different EEG-based measures of consciousness (or degrees thereof) have been employed to investigate neural dynamics during these conditions (Nilsen et al., 2020), in addition to measures based on other techniques.

Crucially, in such studies it has typically been assumed that (or phrased as if) certain brain states, particularly coma, deep slow-wave sleep, and deep general anesthesia, are genuinely unconscious, or that they have significantly reduced level/degree of consciousness (e.g., Crick and Koch, 1990; Casali et al., 2013; Oizumi et al., 2014; MacDonald et al., 2015; Nieminen et al., 2016; Siclari et al., 2017; Juel et al., 2018; Casey et al., 2022). These explicit or implicit assumptions are often based on lack of complex behavior/communication during the state, no recollection of any experience after returning to wakefulness, and/or marked differences in neural dynamics relative to wakefulness (e.g., Crick and Koch, 1990; Searle, 1998; Ferrarelli et al., 2010; Gosseries et al., 2011; Casali et al., 2013; Oizumi et al., 2014; Schartner et al., 2015, 2017; Bonhomme et al., 2019). From a common-sense and pragmatic point of view, these assumptions may seem perfectly reasonable, but are these inferences and assumptions sound? Even if we ignore the epistemological issues discussed earlier, there are empirical reasons to re-examine our categorizations of certain states as states of unconsciousness:

1) **Behavioral signs of (un-)consciousness can be unreliable:** It is of course well known that any behavioral assessment of the degree of (un-)consciousness based on responsiveness to sensory stimuli (e.g., as captured by the Glasgow Coma Scale; Sternbach, 2000, or Richmond Agitation Sedation Scale; Sessler et al., 2002) can be confounded by various sensory or motor deficits. For example, when surgical anesthesia is combined with muscle relaxants, motor responses are prevented. However, by using the isolated forearm technique (IFT), which permits motor reporting by bypassing the neuromuscular blockade, it has been shown that participants undergoing levels of general anesthesia adequate for surgery can still give complex behavioral responses to commands (e.g., “squeeze my hand if you can hear me”) (Russell, 2006; Sanders et al., 2012, 2017; Gaskell et al., 2017; Linassi et al., 2018). This suggests that a level of consciousness sufficient for some voluntary actions (even without postoperative recall) is often maintained in general anesthesia (according to some estimates, IFT responses can be seen in as much as ∼44% of cases^5^; Pandit et al., 2015). For this reason, various neurological measures have been developed to be independent of behavioral inference (e.g., Sleigh et al., 1999; Bekinschtein et al., 2009; Casali et al., 2013; Schartner et al., 2015). However, some such measures have been shown to not detect capacity for behavioral response to commands using the IFT paradigm (Russell, 2006; Gaskell et al., 2017), even when only applying neuromuscular blockade without anesthetics (Schuller et al., 2015). Even if complex motor responses cannot be achieved, such as in certain DOCs, it is in some cases possible to detect volitional behavior through brain computer interfaces, which can then be used as a means of communication (Owen et al., 2006; Monti et al., 2010), although this requires certain stimulus-evoked neural responses to be similar to those in wakefulness^6^ (Gibson et al., 2014). On the other hand, it has been argued that even the presence of complex behavior is not always a reliable indicator of conscious experience (inferred from subjective report), as suggested e.g., by blind sight experiments (Stoerig and Cowey, 1997, but see Phillips, 2021, for an alternative view^7^) or sleepwalking (Oudiette et al., 2009).
2) **Subjective reports of (un-)consciousness can be unreliable:** Subjective retrospective reports (e.g., as used in experiments with intermittent awakening from sleep to probe dreaming; Noreika et al., 2009; Siclari et al., 2018; Aamodt et al., 2021; Casey et al., 2022) are confounded by unreliable recall. For example, propofol and many other anesthetic agents are found to be highly amnesic (Veselis et al., 2008), causing dreams to fade quickly from memory. Thus, obviously, a lack of retrospective report, or report of no experience, can be confounded by a lack of recall (Sanders et al., 2017). This is well known from, e.g., retrograde amnesia induced by scopolamine (Rush, 1988) and anterograde amnesia following hippocampectomy (Dossani et al., 2015). Further, the capacity of iconic memory (sensory memory) has been shown to be much higher than what can be retrospectively reported (Sligte et al., 2008), and this has been suggested to cause phenomenological overflow (Block, 2007). Inherent biases in reporting vague experiences might also mask their presence, as has been argued for blindsight (Phillips, 2021). Finally, even positive reports might be unreliable for many reasons (for a discussion see Schwitzgebel, 2008), including a common tendency to confabulate immediately after waking from sleep or anesthesia (Rosen, 2013).
3) **States presumed to be unconscious are not always devoid of experience:** Careful studies have provided evidence that dreaming occurs surprisingly often in deep non-REM slow-wave sleep stages (Suzuki et al., 2004; McNamara et al., 2010; Windt et al., 2016; Siclari et al., 2017, 2018), as well as during general anesthesia (Käsmacher et al., 1996; Brandner et al., 1997; Leslie et al., 2007, 2009; Eer et al., 2009; Noreika et al., 2011; Leslie, 2017). However, these conditions have often been termed “dreamless” and “unconscious” (e.g., Tononi and Massimini, 2008; MacDonald et al., 2015; Nieminen et al., 2016). Specifically, studies show that the proportion of awakenings from deep non-REM sleep (stage 2–4) that yield immediate reports of dreaming varies from 20 to 70% (Nielsen, 2000; McNamara et al., 2010), and from 11 up to 74% in general anesthesia (Wilson et al., 1975; Leslie et al., 2007; Noreika et al., 2011; Gyulaházi et al., 2015). The reasons for the varying proportion of awakenings with a dream report might be due to methodology (e.g., different delays between awakening and report; Leslie et al., 2007; or how reports are prompted; Stickgold et al., 2001), anesthetic depth (Leslie, 2010), and individual differences such as the propensity to remember dreams in general (Hejja and Galloon, 1975; Eer et al., 2009). For example, a study (Hejja and Galloon, 1975) of dreaming during ketamine anesthesia (which often induces vivid dreams and neural dynamics similar to REM sleep; e.g., Cheong et al., 2011; Sarasso et al., 2015), found that only those who frequently reported dreaming at home also did so following ketamine anesthesia. Similar results were found for propofol anesthesia (Eer et al., 2009). In general, while these observations might be confounded by confabulation or recall of experiences outside of anesthesia, the burden of proving this falls on the claimant. Moreover, the widely varying frequency of dream reports after awakenings from deep sleep or anesthesia, might suggest that more systematic and thorough methods could have obtained reports even more frequently.

In summary, to be able to empirically study the mechanisms enabling conscious experience, requires at least some examples of known changes in degree or presence of consciousness. However, we argue that some of the most widely used current assumptions about such changes are problematic. Specifically, careful experiments have revealed that complex behavior (in the form of volitional communication) and retrospective reports of some kinds of experience (sometimes even vivid and complex) can be obtained from certain conditions more often than our assumptions about the state of consciousness in these conditions would dictate. However, since retrospective reports following anesthesia or sleep can be unreliable due to recall failure and confabulation, it is uncertain to what extent such reports can be trusted. While we do not argue that consciousness never disappears or that there are no brain states that are most correctly described as unconscious (e.g., brain death or catastrophic brain injury that show similar pathological signs of severely disrupted brain activity, such as isoelectric EEG), we do argue that the terminology used when describing various states has implications for clinical practice and basic research.

## Implications

While many of the above empirical points have been raised before (e.g., Hudetz, 2008; Sanders et al., 2012, 2017; Windt et al., 2016), there are still reasons to critically discuss these issues. For example, although it is well known that there are often reports of dreaming following general anesthesia (e.g., Leslie et al., 2009; Noreika et al., 2011; Sanders et al., 2012), many authors still describe general anesthesia as a state of “unconsciousness” (see Supplementary material for some examples). In the last decade alone, 292 papers have been published using the words “unconsciousness” and “anesthesia” in the title or abstract, and 104 papers with “sleep” (with many more using similar wording in the body text), often implying that general anesthesia or deep slow-wave sleep are states of “unconsciousness” (Pubmed search 02.05.2022). While this use of terms may be more appropriate in some contexts than others, it increases the problem of heterogeneous definitions and operationalizations, making it difficult to review the literature. Furthermore, common general differences between wakefulness and states such as deep slow-wave sleep, general anesthesia, and certain DOCs, may be viewed as features associated with loss of consciousness requiring theoretical explanation (e.g., items 1–3 in Table 1 in Seth et al., 2006). However, it has been argued that this may lead to circular reasoning, where theories aiming to explain such features are validated against our “common sense intuition” about consciousness in the very same cases in which the features were detected (Revach and Salti, 2021). While any given theory might not explicitly draw from research assuming the traditional, common sense differentiation between unconscious and conscious states, the theory may be implicitly influenced by this tradition.^8^ Thus, assuming that deep slow-wave sleep, general anesthesia, and certain DOCs, are unconscious states, might lead theoretical development astray. Finally, if we consider as “fully unconscious” states that actually contain frequent dreaming or “other kinds” of experiences, we may miss opportunities to study such “other kinds” of conscious experience (as may be found in deep sleep; Windt et al., 2016).

On the other hand, avoiding our intuitive categorizations of conscious/unconscious states (and the research enabled by their use) has clinical consequences. For example, the question of whether a DOC patient is conscious or not has direct relevance for ongoing and future treatment, and is important for the patient’s caretakers and next of kin. Moving away from the term “unconscious” might then weaken the support for clinical tools developed to help diagnose DOC patients. Therefore, criticisms of current use of the concept and term “unconsciousness” should avoid hasty conclusions, else risking “throwing the baby out with the bathwater.”

## Possible solutions

The primary purpose of this perspective is to suggest that, given the evidence of disconnected or covert experiences occurring surprisingly often in states commonly labeled as “unconscious” (and the multiple confounds in inferring the absence of consciousness), one should be more cautious in how one uses the concept and term “unconsciousness”, primarily, in basic consciousness research.

However, if states like general anesthesia, slow-wave sleep, and certain DOCs, are no longer valid as contrastive paradigm cases of unconsciousness in consciousness science, it seems hard to identify mechanisms necessary and sufficient for consciousness. We believe that current approaches that focus more on “degree of similarity” to normal wakefulness, or on how confident we can be that there is some conscious experience (and perhaps of what), can avoid the issues discussed above. Here we outline three such approaches.

1. **Pragmatic approach:** Rather than working from prior assumptions, we can classify states based on how they differ from normal wakefulness, or based on the confidence we have that they might support any form of consciousness. Most studies following a “state-based” approach (i.e., contrasting system level global changes) already do this when using wakefulness in healthy subjects as a reference (control) condition. Accordingly, several measures used in the clinic are more appropriately described as “positive markers,” that is, markers capturing the “degree of similarity” to wakefulness in healthy subjects (e.g., King et al., 2013; Casarotto et al., 2016; Demertzi et al., 2019). In other words, such markers help build confidence in the inference that a subject is conscious. While researchers using such markers are more or less explicitly clear on this, it is still too common to employ the term “unconsciousness” when referring to states different *enough* from wakefulness when, we argue, one should rather be explicitly agnostic about the ground truth in these cases. This approach entails avoiding generalizing across various states and conditions under the assumption that they share the underlying property of unconsciousness. Alternatively, one can sidestep the issue by clearly defining consciousness (or particularly the absence thereof) in more operational terms (as in e.g., Siclari et al., 2018, Sarasso et al., 2021). While this may be a step in the right direction, it depends on the field agreeing on definitions and operationalizations to avoid confusion, which still seems some ways off (Francken et al., 2022).
2. **Descriptive approach:** Extending the pragmatic approach, we can systematically describe states and conditions functionally, behaviorally, cognitively, and physiologically (e.g., as in Sanders et al., 2012), to obtain a more complete and nuanced picture of the states, while remaining agnostic as to whether consciousness is truly present/absent (in the Nagelian sense) (see also Bayne et al., 2016, 2020; Engemann et al., 2018; Walter, 2021). For example, a moderate level of propofol anesthesia could be characterized as a state of partial responsiveness and partially connected awareness (IFT shows command following), partial/occasional recall (it is possible to obtain some dream reports), impaired explicit memory formation (ability to report dreams quickly declines after awakening), and partial capacity for communication (through IFT). Then, such properties can be empirically determined and compared between different states and conditions. For example, vivid “dream” reports (much like those following REM sleep) are currently thought to be more common after ketamine anesthesia than other anesthetics (Leslie, 2010), and the propensity of dream reports has been observed to increase with sedation depth for propofol, but not for other anesthetics (Eer et al., 2009). The latter study also illustrates which kinds of questions a more descriptive approach could incentivize, e.g., sedation-depth/report relationships.
Further, characterizing states and conditions in this manner allows: (1) large scale contrasting and comparison of the outcome of various neurological measures between different states (e.g., as in Gordillo et al., 2020; Nilsen et al., 2020); (2) studying memory, confabulation, perception, meta-cognition, etc., within various states in order to build a basis for estimation of reliability of subjective reports and the inferences that can be drawn from such reports (e.g., evidence of at least implicit learning in anesthesia; Ghoneim and Block, 1997); and, (3) mapping the heterogenous causes and effects of various states and conditions allowing for more nuanced treatment of patients. For example, Gibson et al. (2014) show how individual DOC patients differ in volitional mental imagery ability and where in the cortex such imagery might be detected. Such individual variation might otherwise be lost if a single axis of conscious-unconscious or cognitive capacity is assumed.
Finally, the specific characterizations of various states, as exemplified above, can then be updated accordingly in a more iterative fashion as new knowledge is acquired.
3. **Predictive approach:** In order to support a more pragmatic and descriptive approach, one may focus on predicting what contents are experienced by subjects in specific situations based on their neural activity, i.e., developing and applying “mind-readers.” This approach may help build confidence in the validity of subjective reports of phenomenological experience in wakefulness. From there, one can gradually approach states and conditions in which we have reason to doubt subjective reports. Although the field is still in its infancy, there have been several advances in this regard, for example decoding dreams (Horikawa et al., 2013; Smith, 2013; Horikawa and Kamitani, 2017), movie watching (Huth et al., 2016; Nishida and Nishimoto, 2018), speech (Wang et al., 2021), and other sensory modalities (Hatamimajoumerd et al., 2022). This approach is similar to solving the “real” problem of consciousness (Seth, 2016). However, a “mind-reader” faces similar criticisms as that of “consciousness-meters” (Latham et al., 2017) in that it’s in principle impossible to validate in general, but especially in states and conditions where reports are lacking or unclear. Furthermore, decodable neural activity is not necessarily associated with conscious processing *per se* (Lepauvre and Melloni, 2021). On the other hand, a predictive approach can of course be beneficial beyond the study of consciousness; e.g., objective indications that someone experiences pain can be of major importance in the clinic.
For more generalized forms of “mind-readers,” one might identify basic dimensions of conscious experience (e.g., as in Bayne et al., 2016; Fazekas and Overgaard, 2016), e.g., differences in abstraction level of percepts, the range of possible qualia, or the “richness”/ “vividness”/“resolution” of those experiences or experience as a whole. Insofar as these concepts can be mapped reliably to behavior, subjective reports, or neuroimaging, with testable outcomes, this approach might be used for measures that track general properties of representational content (e.g., Siclari et al., 2018; Farnes et al., 2020; Aamodt et al., 2021). While this approach doesn’t solve the issue raised here—e.g., it is unclear whether a “less vivid” experience is “less conscious”—it can allow for investigation into experiential states that are far removed from normal wakefulness.

## Conclusion

In sum, we argue that there are strong reasons to take a step back and critically re-examine our “traditional” assumptions about different states of consciousness. Although such issues have been discussed for some time, there seems to remain a tendency to still use outdated assumptions and terminology, e.g., that general anesthesia and slow-wave sleep entail full or nearly full unconsciousness. We suggest that focusing on approaches that provide a more pragmatic and nuanced characterization of different states and conditions might be less controversial, promote clarity, and can help build a stronger foundation for future studies.

## Data availability statement

The original contributions presented in this study are included in the article/Supplementary material, further inquiries can be directed to the corresponding authors.

## Author contributions

ASN wrote the first draft and did most of the work on the manuscript. All authors contributed to the ideas and arguments, writing, and revision of the manuscript.

## Footnotes

1 A “phenomenology-first approach” (infer objective properties from subjective properties) has been proposed to sidestep the issue of inference of subjective properties from objective properties (Ellia et al., 2021). However, this method has been criticized for being tautological or unfalsifiable (Doerig et al., 2019; Kleiner and Hoel, 2021), but see (Negro, 2020) for a defense.

2 Changes in “brain states” are here taken to include changes at the micro and/or macro levels.

3 If the central research question is to understand why, how, or when, a system is conscious or not, one needs an exemplar of a system or state that is in fact not conscious (there is “not like anything to be”). This is similar to the hard problem of consciousness (Chalmers, 1995).

4 Unless otherwise stated, general anesthesia refers to forms of anesthesia, such as propofol, xenon, thiopental, and iso-, sevo-, and des-flurane, that induce slow-wave activity (SWA) in the EEG. In contrast, ketamine does not induce SWA and is associated with reports of vivid dreams (Leslie, 2010), and common EEG-based anesthesia monitors are not indicative of sedation depth (Hirota, 2006), which has given ketamine anesthesia a special case in consciousness research.

5 Anesthetic regimens that use IFT during surgery often deepen sedation if detecting a sufficiently complex IFT response during or prior to surgery, as well as usually employing a mix of analgesics, muscle relaxants, and anesthetics. This can skew dose-response estimates. Further, while increased anesthetic concentration does seem to reduce degree of IFT responsivity, it is debatable whether this marks a gradual decline in consciousness, or a move into different “states” of consciousness (e.g., dysanesthesia) (Pandit et al., 2015).

6 In Owen et al. (2006), the presence of neural activation in response to spoken sentences, similar to that of healthy participants, encouraged the researchers to attempt using this for communication.

7 The “classic” interpretation of blindsight is no phenomenology but complex behavior. Phillips (2021), however, proposes that there is phenomenology and behavior, but normally no report. Specifically, Phillips argues that blindsight is severely degraded vision, but due to negative response bias is not reported as such. This supports our argument that what we do or do not say or do does not necessarily reflect our conscious experience.

8 Integrated information theory (IIT) specifically aims to start from first principles and thus avoid the issue discussed here (although the mentioned assumptions are used as supporting arguments; e.g., Tononi, 2012; Oizumi et al., 2014). In fact, IIT, and several other structural theories (Doerig et al., 2021), imply that the entire brain (or some parts of it) is never fully unconscious, or that there might be phenomenological experiences locally which are not accessible for report later (or currently) (Lamme, 2010). IIT does, however, imply a “graded” construct in its theoretical measure “Phi” which has inspired empirical measures that broadly support the theory’s predictions (Casali et al., 2013; Sarasso et al., 2021). However, in order to validate the theory, one must assume that states like deep slow-wave sleep or general anesthesia are states of, at least, reduced consciousness, which leads to falsification problems (Doerig et al., 2019; Kleiner and Hoel, 2021).
