# Supplementary material for: Are we really unconscious in “unconscious” states? Common assumptions revisited
Source: Front Hum Neurosci. 2022 Oct 5;16:987051. doi: 10.3389/fnhum.2022.987051 (PMC9581328; doi:10.3389/fnhum.2022.987051)
Supplement: Supplementary file 1 [file Data_Sheet_1.pdf]

Appendix

To provide some typical examples of the general issue that we are discussing in our paper, we here give some citations from the literature (in chronological order) about states of presumed unconsciousness, taken from articles referenced in the main text. We do not imply that these citations indicate that the authors were not aware of the issues discussed in this paper or think that the brain states they refer to are necessarily examples of complete unconsciousness. In fact, personal communications with several of the authors indicates that they are well aware of these issues. However, if taken literally, the exact wording of these citations often do not seem to clearly reflect this awareness, but may rather seem to refer more to traditional “common-sense” views that seem incompatible with more recent evidence, e.g. reports of frequent dreaming during anesthesia and slow-wave sleep. It is important to note that in some of the papers referenced below, the terms used are explicitly or implicitly operationalized (e.g. unconsciousness = unresponsive states with lack of retrospective report) or treated with more nuance (e.g. states can be more or less unconscious, or general anesthesia being a state of unconsciousness at least parts of the time).

“It seems likely that the essential features of consciousness are probably not usually present in slow-wave sleep, nor under a deep anaesthetic. Rapid Eye Movement (REM) or dreaming sleep is another matter. It seems to us that a limited form of consciousness often occurs in REM sleep.” (Crick & Koch, 1990)

“We hypothesized that a similar breakdown of cortical effective connectivity may underlie loss of consciousness (LOC) induced by pharmacologic agents [midazolam].” (Ferrarelli et al., 2010)

“PCI discriminates between consciousness and unconsciousness in healthy individuals.” and “[...] unconscious (sleep- and anesthesia-mediated loss of consciousness).” (Casali et al., 2013)

“Why does consciousness fade early in sleep, although the brain remains active? Why is it lost during generalized seizures, when neural activity is intense and synchronous?” (Oizumi et al., 2014)

“As an experimental paradigm, anesthesia allows consciousness to be reliably and reproducibly abolished in healthy individuals.” (MacDonald et al., 2015)

“These findings are theoretically relevant, confirming the prediction that loss of consciousness during anesthesia is tied to a reduction of brain complexity [...]” (Sarasso et al., 2015)

“By contrast, when subjects are considered unconscious and do not report any dreams, like typically in non-REM (NREM) sleep early in the night, in propofol-, xenon-, or midazolam-induced anesthesia and in the unresponsive wakefulness syndrome/vegetative state, [...]“ (Nieminen et al., 2016)

“[...] our measures provide direct evidence for a breakdown of differentiation between regions and diversity of brain states explored, during states of unconsciousness [NREM sleep] [...]” (Schartner et al., 2017)

“Sleep is the only time in which consciousness fades under normal physiological conditions: subjects awakened from sleep, especially early in the night, report that they were not experiencing anything up to 30% of the time. At other times, subjects awakened from sleep report dreams — a stream of vivid experiences that occur despite being immobile, unresponsive, and largely disconnected from the environment. Thus, unlike wakefulness, sleep can be associated with either the presence or absence of conscious experiences.” (Siclari et al., 2018)

“The search for such tools should be guided by scientific findings related to changes in brain activity induced by loss of consciousness in general, and general anesthesia in particular. Over the past decades, studies have revealed many reproducible changes in electroencephalography (EEG) activity related to anesthesia-induced loss of consciousness, […]” (Juel et al., 2018)

“For instance, the default-mode network (DMN) is more or less preserved during unconscious states [general anesthesia]” (Northoff & Lamme, 2020)

“These findings may enable novel monitors of the anaesthetic state that can distinguish sensory disconnection and unconsciousness [in dexmedetomidine, sleep, and propofol], and these may provide novel insights into the biology of arousal.” (Casey et al., 2022)

References

Casali, A. G., Gosseries, O., Rosanova, M., Boly, M., Sarasso, S., Casali, K. R., Casarotto, S., Bruno, M.-A., Laureys, S., Tononi, G., & Massimini, M. (2013). A theoretically based index of consciousness independent of sensory processing and behavior. *Science Translational Medicine*, *5*(198), 198ra105. https://doi.org/10.1126/scitranslmed.3006294

Casey, C. P., Tanabe, S., Farahbakhsh, Z., Parker, M., Bo, A., White, M., Ballweg, T., Mcintosh,

A., Filbey, W., Saalmann, Y., Pearce, R. A., & Sanders, R. D. (2022). Distinct EEG signatures differentiate unconsciousness and disconnection during anaesthesia and sleep. *British Journal of Anaesthesia*, *128*(6), 1006–1018. https://doi.org/10.1016/j.bja.2022.01.010

Crick, F., & Koch, C. (1990). Towards a neurobiological theory of consciousness. *Seminars in the Neurosciences*, *2*, 263–275. https://authors.library.caltech.edu/40352/

Juel, B. E., Romundstad, L., Kolstad, F., Storm, J. F., & Larsson, P. G. (2018). Distinguishing Anesthetized from Awake State in Patients: A New Approach Using One Second Segments of Raw EEG. *Frontiers in Human Neuroscience*, *12*, 40. https://doi.org/10.3389/fnhum.2018.00040

MacDonald, A. A., Naci, L., MacDonald, P. A., & Owen, A. M. (2015). Anesthesia and neuroimaging: investigating the neural correlates of unconsciousness. *Trends in Cognitive Sciences*, *19*(2), 100–107. https://doi.org/10.1016/j.tics.2014.12.005

Nieminen, J. O., Gosseries, O., Massimini, M., Saad, E., Sheldon, A. D., Boly, M., Siclari, F., Postle, B. R., & Tononi, G. (2016). Consciousness and cortical responsiveness: a within-state study during non-rapid eye movement sleep. *Scientific Reports*, *6*, 30932. https://doi.org/10.1038/srep30932

Oizumi, M., Albantakis, L., & Tononi, G. (2014). From the phenomenology to the mechanisms of consciousness: integrated information theory 3.0. *PLoS Computational Biology*, *10*(5), e1003588. http://journals.plos.org/ploscompbiol/article?id=10.1371/journal.pcbi.1003588

Siclari, F., Bernardi, G., Cataldi, J., & Tononi, G. (2018). Dreaming in NREM Sleep: A High-Density EEG Study of Slow Waves and Spindles. *The Journal of Neuroscience: The Official Journal of the Society for Neuroscience*, *38*(43), 9175–9185.

https://doi.org/10.1523/JNEUROSCI.0855-18.2018

Ghoneim, M. M., & Block, R. (1997). Learning and memory during general anesthesia: an update. *The Journal of the American Society of Anesthesiologists*, *87*(2), 387-410.

Stickgold, R., Malia, A., Fosse, R., Propper, R., & Hobson, J. A. (2001). Brain-mind states: I. Longitudinal field study of sleep/wake factors influencing mentation report length. *Sleep*, *24*(2), 171-179.

Pinker, S. (1997). How the mind works (Vol. 524). Norton: New York.

Varela, F. J., Thompson, E., & Rosch, E. (2017). The embodied mind, revised edition: Cognitive science and human experience. *MIT press*.
